# The value of NGS-based multi-gene testing for differentiation of benign from malignant and risk stratification of thyroid nodules

**DOI:** 10.3389/fonc.2024.1414492

**Published:** 2024-11-12

**Authors:** Mingjian Fei, Dongdong Ding, Xuanyi Ouyang, Wenyan Shen, Fenglan Zhang, Bo Zhang, Lan Qin

**Affiliations:** ^1^ Department of Pathology, The Second Affiliated Hospital of Jiaxing University, Jiaxing, China; ^2^ Center for Clinical Genetics and Genomics, Dian Diagnostics Group Co., Ltd., Hangzhou, China; ^3^ Department of Ultrasonic Diagnosis, The Second Affiliated Hospital of Jiaxing University, Jiaxing, China; ^4^ Department of Thyroid Surgery, The Second Affiliated Hospital of Jiaxing University, Jiaxing, China

**Keywords:** thyroid nodules, next-generation sequencing, fine-needle aspiration, molecular diagnostics, risk stratification

## Abstract

**Background:**

Fine-needle aspiration (FNA) biopsy is typically used in conjunction with cytopathologic evaluation to differentiate between benign and malignant thyroid nodules. Even so, the cytology results for 20-30% of thyroid nodules are indeterminate. This study sought to evaluate the usefulness of next-generation sequencing (NGS)-based multi-gene panel testing for risk stratification and the differentiation of benign from malignant thyroid nodules.

**Methods:**

Thyroid nodule samples were obtained from a cohort of 359 patients who underwent FNA. An NGS-based multi-gene panel testing was conducted for these samples, in which single-nucleotide variants (SNVs) and small insertion/deletions (InDels) can be detected in 11 genes and fusion events can be identified in 5 genes. Surgical resection was conducted for 113 patients (113/359), and then histopathology results were obtained.

**Results:**

In comparison to cytology alone, the diagnostic sensitivity of NGS combination cytology increased from 0.7245 (95% CI: 0.6289-0.8032) to 0.898 (95% CI: 0.8223-0.9437); the associated AUC was 0.8303 (vs. Cytology AUC: 0.7622, *P* < 0.001). *BRAF* ^V600E^ was identified in 136 patients, of whom 79 underwent surgery and were diagnosed with papillary thyroid carcinoma (PTC) pathologically. *TERT* promoter mutations or *BRAF*/*RAS* co-mutations with other genes were identified in 5 patients, while 4 patients were diagnosed with malignant thyroid cancer using the pathological method. *RAS* mutations were identified in 27 patients, while 10 patients underwent surgery, which showed that 3 patients were classified as PTC and 7 cases were benign. In addition, 4 *RET* fusions, 1 *RET* activation mutation, and 3 *TP53* inactivation mutations were identified in the remaining 8 patients who have not undergone surgery. Negative genetic test results or variants with uncertain significance were identified in 183 patients. Among these patients, 12 malignant thyroid tumors, including 11 PTC and 1 MTC, were diagnosed in 20 patients who received surgery.

**Conclusion:**

Thyroid nodules coupled with *BRAF* ^V600E^, *TERT* promoter variants, *BRAF*/*RAS* co-mutations with other genes, *RET* fusions, and *RET* activating mutations were classified as high-risk. Nodules with *RAS* mutations (*NRAS*, *KRAS*, *HRAS*) and *TP53* inactivating mutations were considered to be in the intermediate-risk group, while those with non-pathogenic mutations (negative and variants of uncertain significance) were placed in the low-risk group. When combined with cytopathology, NGS increases the sensitivity of diagnosing benign and malignant thyroid nodules, and the reference is useful for patient risk stratification.

## 1 Introduction

Thyroid cancer (TC) is an endocrine illness that includes three primary forms: differentiated thyroid cancer (DTC), medullary thyroid carcinoma (MTC), and anaplastic thyroid carcinoma (ATC). According to the latest available data, there were 39,079 new TC diagnoses in 2019 with an age standard incidence rate (ASIR) of 2.05/100,000 and 7,240 deaths with an age standard mortality rate (ASMR) of 0.39/100,000 (1). In China, the ASIR was 3.21/105 in 2005 and increased to 9.61/105 in 2015, whereas the ASMR was 0.30/105 in 2005 and rose to 0.35/105 in 2015 (2). DTC patients had an overall excellent prognosis, with a 5-year survival rate of 98.5% when given appropriate treatment (3). High-resolution ultrasonography was used to detect thyroid nodules, and the results showed a prevalence of 20-76%, in which 10-15% of patients harbored cancerous thyroid nodules (4, 5). Thyroid nodules require medical attention because they have the potential to become malignant.

When combined with cytopathology and taking the nodule’s size and ultrasound appearance into consideration, a fine-needle aspiration (FNA) biopsy is the most widely used and cost-effective diagnostic technique for distinguishing benign from malignant nodules. According to recent research, the benign and malignant nature of thyroid nodules in a sizable fraction of patients was consistent with their FNA cytopathology report, even though a tiny percentage of samples showed cytological indeterminacy. The aforementioned conditions comprise 2-18% of nodules with atypia of undetermined significance/follicular lesion of undetermined significance, 2-25% with follicular neoplasm/suspicious for follicular neoplasm, and 1-6% with suspicious for malignancy (6). Indeterminate thyroid nodules can now be more accurately diagnosed when combining cytologic diagnosis with molecular testing.

In 2014, the Cancer Genome Atlas Research Network (TGCA) conducted a study on the molecular profiles of papillary thyroid cancers (PTCs), which showed two distinct molecular profiles: *BRAF* ^V600E^-like and *RAS*-like (7). The widely accepted molecular classifications are as follows: *BRAF* ^V600E^ -like included the *BRAF* ^V600E^ mutation and *RET* and *BRAF* fusions; *RAS*-like included RAS family (*KRAS*, *HRAS*, and *NRAS*) mutations, *BRAF* ^K601E^ mutation, *EIF1AX* mutations, and *PPARG* and *THADA* fusions (8). A study based on 458 Chinese patients with thyroid cancer reported that the prevalence of *BRAF* driver mutations is 76.0%, followed by *RET* rearrangements (7.6%), while the prevalence of *RAS* mutations is only 4.1% (9). Additional molecular profiles have also been thoroughly investigated. These include promoter mutations in *TERT*, which are independently predictive of DTC-related death and strongly associated with high tumor aggressiveness (10). *BRAF*/*RAS* co-mutations with other genes (e.g., *TERT*, *PIK3CA*, and *TP53*) indicate an increasing risk of malignancy in thyroid cancer (6).

According to current guidelines, molecular testing may be employed to supplement the malignancy risk assessment of thyroid nodules. In this study, we applied a molecular test project including 11 genes for thyroid FNAs. Based on the molecular profiles detected in thyroid nodules, we aimed to evaluate the value of our next-generation sequencing (NGS)-based multi-gene testing panel for the differentiation of benign from malignant and the risk stratification of thyroid nodules.

## 2 Materials and methods

### 2.1 Patients

359 thyroid nodule samples from patients who underwent FNA operations at the Second Affiliated Hospital of Jiaxing University between May 2022 and December 2023 were collected both retrospectively and consecutively. NGS-based panel testing is recommended for patients with (1) thyroid nodules with malignant features on conventional ultrasound: solid, hypoechogenicity, microcalcification, unclear margins, vertical growth, or taller-than-wide shape; and (2) thyroid nodules that have grown too rapidly (more than 50% increase in size in one year) or have new malignant ultrasound features during follow-up. Patients for whom NGS-based panel testing is not recommended: (1) pure cystic nodules; and (2) those with coagulation abnormalities.

The FNA biopsy samples underwent both cytology and NGS-based multi-gene panel testing simultaneously. 113 patients received surgical resections, and a histopathology assay was conducted. FNA biopsy samples and surgically resected tissues were embedded in paraffin, sectioned, and stained with hematoxylin and eosin. The results were reviewed and reported by two pathologists. The mean time between FNA biopsy and surgical resection in these 113 individuals was 29.6 days, with a median of 19 days. The clinic data, NGS test results, cytological diagnoses, and histological diagnoses were all subjected to a retrospective analysis. This study was approved by the Ethics Committee of the Second Affiliated Hospital of Jiaxing University. All subjects signed informed consent forms.

### 2.2 Next-generation sequencing

DNA for next-generation sequencing was extracted using the Quick-DNA/RNA Microprep Plus Kit (D7005, Zymo Research Corp., USA). The libraries were then established with the Thyroid Cancer Mutation Analysis Panel Kit (Dian Diagnostic, Hangzhou, China), which includes SNV/InDel for 10 genes (coding sequence of *BRAF*, *HRAS*, *KRAS*, *NRAS*, *TP53*, *RET*, *NTRK1*, *NTRK3*, *PAX8*, *THADA*), promote region of the TERT gene, and 5 frequently rearranged genes in thyroid carcinoma (*RET*, *NTRK1*, *NTRK3*, *PAX8*, and *THADA*), according to the standard operating procedure. The libraries were then sequenced on an Illumina NextSeq 500 platform (Illumina, San Diego, CA).

### 2.3 Date analysis

FASTQ sequencing files were aligned to the human reference genome (UCSC hg19; Feb 2009 release) with Burrows-Wheeler Aligner Tool (BWA, version 0.7.17-r1188). STAR-Fusion (version 1.12.0) and Arriba software (version 2.1.0) were used to detect gene fusions. The candidate gene fusion transcripts were reviewed and visualized in Integrative Genome Viewer (IGV, Broad Institute, version 2.13.0). Given the unavailability of germline sequencing data, we employed a rigorous filtering strategy to eliminate potential germline variants. Variants present in one or more population databases (ESP, 1000Genome, ExAC, gnomAD) with MAF ≥ 1% were excluded. Variants classified as “benign” or “like benign” in the ClinVar database were also excluded.

### 2.4 Statistical analysis

We built linear regression to determine if the NGS combined with cytology can predict the benign and malignant thyroid nodules. Sensitivity (Sen), specificity (Spe), positive predictive value (PPV), and negative predictive value (NPV) were analyzed with reference to the receiver operating characteristic curve (ROC). The area under the receiver operating characteristic curve (AUC) was plotted to assess the diagnostic performance. Statistical analyses were performed using VassarStats, an open-source statistical tool. Studies have shown that 95% confidence intervals (CIs) provide good coverage over time. And our research spanned 18 months, so the confidence interval was reported as a two-sided 95%.

## 3 Results

### 3.1 Basic information of the patients included in this study

359 FNA biopsy samples of thyroid nodules were analyzed in this work, and an NGS-bases multi-gene analysis was ordered to detect genomic alterations. The position and size of the thyroid nodule were determined by ultrasonography, and the thyroid cytopathologic diagnosis was established based on the Bethesda System. The final diagnosis was based on histopathology (if the patient underwent surgery). The clinical data of the patients is shown in **Table 1**. The age of patients ranged from 19 to 79 years (mean 46 years), and the ratio of females to males was 2.55:1. All samples have previously undergone cytopathology. 33 (9.19%) samples were classified as non-diagnostic (Bethesda I), 149 (41.50%) as benign (Bethesda II), 47 (13.10%) as atypia of indeterminate significance/follicular lesion of indeterminate significance (AUS/FLUS, Bethesda III), 7 (1.95%) as follicular neoplasm/suspicious for follicular neoplasm (FN/SFN, Bethesda IV), 17 (4.74%) as suspicious for malignancy (SUSP, Bethesda V), and 123 (29.53%) as malignant (Bethesda VI). Following an 18-month follow-up, 113 out of 359 patients received surgical resection, in which 98 patients were diagnosed as having malignant thyroid tumors, including 95 PTC, 1 FTC, 1 MTC, and 1 differentiated high-grade thyroid carcinoma (DHGTC).

**Table 1:** Demographic and clinical characteristics of this study.

	variable	number	%
**Total**	Patients	359	–
	FNAs	359	–
**Sex**	Male	101	28.13%
	Female	258	71.87%
**Age, years**	19-29	38	10.58%
	30-39	92	25.63%
	40-49	75	20.89%
	50-59	88	24.51%
	60-69	44	12.26%
	≥70	22	6.13%
**Bethesda**	I	33	9.19%
	II	149	41.50%
	III	47	13.09%
	IV	7	1.95%
	V	17	4.74%
	VI	106	29.53%
**Surgical pathology**	Papillary thyroid carcinoma	95	26.46%
	Follicular thyroid carcinoma	1	0.28%
	Medullary thyroid carcinoma	1	0.28%
	Differentiated high-grade thyroid carcinoma	1	0.28%
	Benign	15	4.18%
	No surgery	246	68.52%

### 3.2 NGS combined with cytology improved the detection rate of malignant nodules

**Figure 1:**
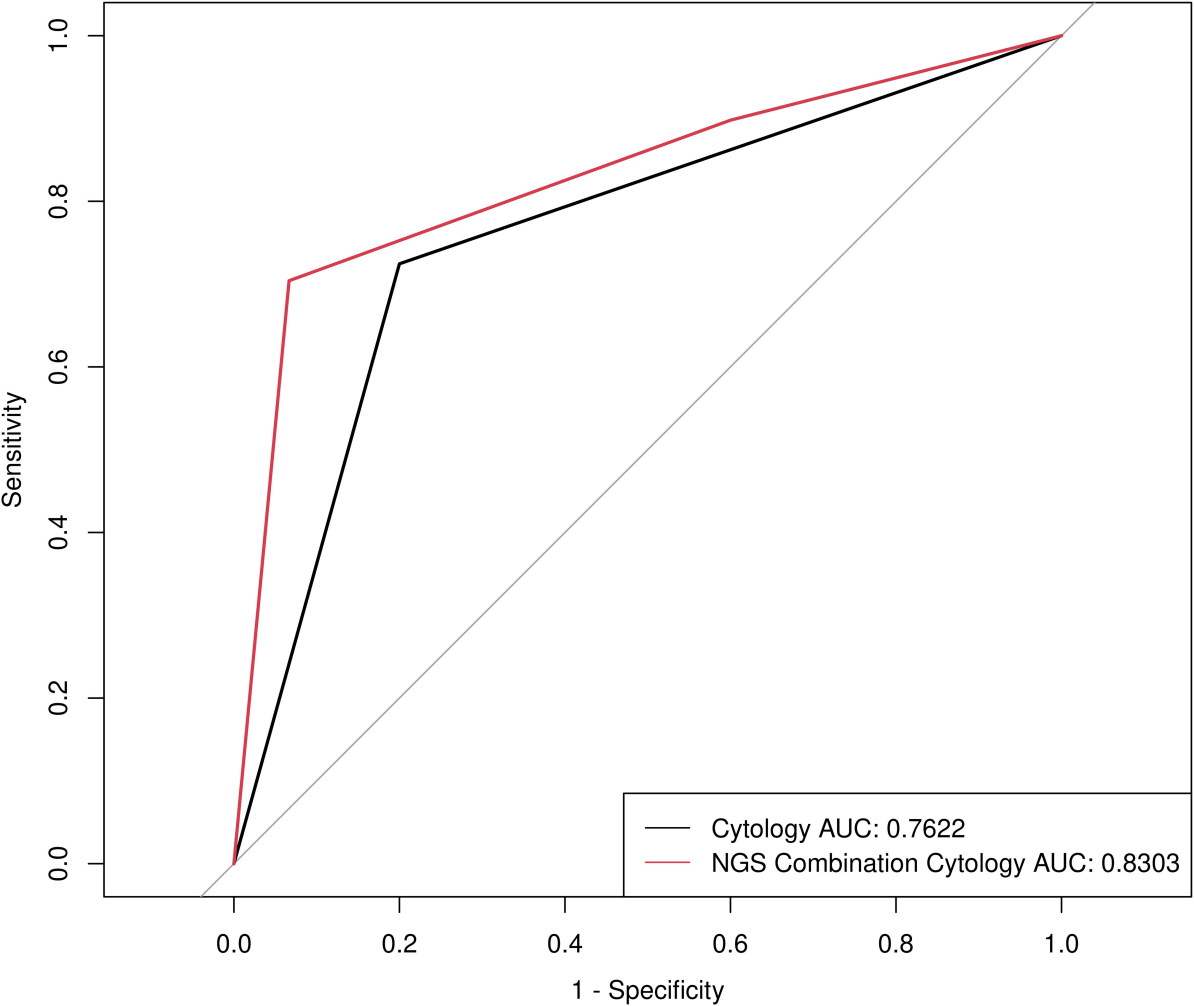
Receiver operating characteristic (ROC) curve for determining benign and malignant thyroid nodules by Cytology and NGS combination Cytology.

To assess the diagnostic efficiency of NGS for malignant nodules, we compared the diagnostic sensitivity and specificity of cytology and NGS combined with cytology. The diagnostic sensitivity of NGS combined with cytology increased from 0.7245 (95% CI: 0.6289-0.8032) to 0.898 (95% CI: 0.8223-0.9437), while the positive predictive value (PPV) and negative predictive value (NPV) showed no statistically significant change (**Table 2**). The patients with *RAS* mutations enrolled in this study were the reason for the significant decrease in specificity, as thyroid nodules with RAS mutations belong to the intermediate-risk category. However, a ROC curve was calculated with an AUC of 0.8303 (*P* < 0.001) (**Figure 1**), indicating an excellent diagnostic performance of the NGS combination cytology.

**Table 2:** Diagnostic efficiency of cytology and NGS combined with cytology.

Cytology				
	Bethesda I to IV	Bethesda V/VI		
Histopathology Malignant	27	71	Sensitivity with 95% CI: 0.7245 (0.6289-0.8032) Specificity with 95% CI: 0.8 (0.5481-0.9295) PPV with 95% CI: 0.9595 (0.8875-0.9861) NPV with 95% CI: 0.3077 (0.1857-0.4642)	
Histopathology Benign	12	3		
NGS Combination Cytology				
	Bethesda I to IV without Pathogenic Mutation	Bethesda V/VI without Pathogenic Mutation or Bethesda I to IV with Pathogenic Mutation	Bethesda V/VI with Pathogenic Mutation	
Histopathology Malignant	10	19	69	Sensitivity with 95% CI: 0.898 (0.8223-0.9437) Specificity with 95% CI: 0.4 (0.1982-0.6425) PPV with 95% CI: 0.9072 (0.833-0.9504) NPV with 95% CI: 0.375 (0.1848-0.6136)
Histopathology Benign	6	8	1	

Sensitivity, specificity, PPV and NPV of diagnosis were based on cytology (Bethesda V/VI are considered malignant) and NGS combined with cytology (Bethesda V/VI and/or pathogenic mutation are considered malignant).

In this study, 113 patients underwent surgical resection, 84 of whom had thyroid nodules <10 mm. In comparison to cytology alone, the diagnostic sensitivity of NGS combined with cytology demonstrated an increase from 0.75 (95% CI: 0.6422-0.8337) to 0.9211 (95% CI: 0.8383-0.9633). It is proposed that the combination of NGS and cytology may significantly enhance the precision of preoperative diagnosis of thyroid nodules with a diameter of <10 mm. Consequently, it can be concluded that the NGS can markedly improve the detection rate of malignant thyroid nodules in comparison to that by cytology.

### 3.3 Diagnostic significance of different molecular characteristics in thyroid nodules

NGS-based multi-gene testing panel was used to analyze the molecular characteristics of 359 FNA biopsy samples of thyroid nodules (**Table 3**). With regard to the 113 patients who had thyroid nodule resection operations and the 246 patients who did not, all Bethesda classifications were covered.

**Table 3:** Molecular features of 359 FNA biopsy samples of thyroid nodules by analysis of NGS based multi-gene testing panel.

Genetic alterations	Bethesda I	Bethesda II	Bethesda III	Bethesda IV	Bethesda V	Bethesda VI	Surgery			No surgery
	(n=33)	(n=149)	(n=47)	(n=7)	(n=17)	(n=106)	Total	Histopathology Malignant	Histopathology Benign	Total
*BRAF* ^V600E^	6	6	17	0	14	93	79	79	0	57
*TERT*	0	0	0	1	0	0	1	1	0	0
*BRAF* ^V600E^+*TP53*	0	0	0	0	0	1	1	1	0	0
*KRAS*+*TP53*	0	0	0	0	0	1	1	1	0	0
*HRAS*+*TERT*	0	1	0	0	0	0	1	1	0	0
*BRAF* ^V600E^+*TERT*	0	0	0	0	0	1	0	0	0	1
*NRAS*	1	7	3	1	1	1	8	3	5	6
*KRAS*	0	8	1	1	0	0	2	0	2	8
*HRAS*	1	1	0	1	0	0	0	0	0	3
*RET* fusion	0	3	1	0	0	0	0	0	0	4
*RET*	0	1	0	0	0	0	0	0	0	1
*TP53*	0	3	0	0	0	0	0	0	0	3
non-pathogenic	25	119	25	3	2	9	20	12	8	163

The most prevalent harmful mutation in those samples was *BRAF* ^V600E^. 136 patients with thyroid nodules carrying *BRAF* ^V600E^ were identified from 359 FNA biopsy samples. Of these, 79 underwent surgery and were pathologically diagnosed as suffering from papillary thyroid carcinoma (PTC), including two nodules with a size ≤10 mm and FNA cytology classified as non-diagnostic results (Bethesda I). 56 patients in this cohort of patients who did not underwent surgery carried *BRAF* ^V600E^, of which 71.43% (n = 40) were suspicious for malignancy or malignant thyroid nodules diagnosed by FNA cytopathology and classified as Bethesda V/VI.

5 patients with thyroid nodules carried molecular characteristics such as *TERT* promoter mutations (n = 1) and *BRAF*/*RAS* co-mutations with other genes (*BRAF* ^V600E^ +*TP53* n = 1, *KRAS*+*TP53* n = 1, *HRAS*+*TERT* n = 1, *BRAF* ^V600E^ +*TERT* n = 1), which are known to indicate an increased risk of malignancy in thyroid cancer. 4 out of these patients underwent surgery, and surgical pathology results indicate malignant nodules, including 2 PTC, 1 DHGTC, and 1 FTC. A rare type of DHGTC was confirmed histopathologically in a patient with a *TERT* promoter variation based on a resected nodule exhibiting ≥5 mitoses per 2 mm2 and/or tumor necrosis. Despite the cytology indicating a classification of Bethesda II, a patient with co-mutations in *HRAS* and *TERT* was identified as having a widely invasive FTC through the surgical pathology method. The prognosis of FTC is generally worse than that of PTC, and widely invasive FTC is the subtype with the worst prognosis.

*RAS* variants were detected in 27 FNA biopsy samples. Of these, the *NRAS* mutations of interest were all exon3 p.Q61X; the *HRAS* and *KRAS* variants found were the common activating mutations p.G12X, p.G13X, and p.Q61X. 10 of these patients received surgical treatment (*NRAS* mutations n = 8, *KRAS* mutations n = 2). Among these 10 patients, 3 were diagnosed with PTC, and 7 had benign conditions. Interestingly, none of the PTCs had harmful *KRAS* mutations; all of them only carried deleterious *NRAS* mutations. It’s probable that harmful *NRAS* variants are more useful for diagnosis than *KRAS* variants.

Further pathogenic variations found in patients who did not undergo surgery include *RET* activating mutations (n = 1), *RET* fusions (n = 3), and *TP53* inactivating mutations (n = 3). Elevated calcitonin in a patient with a *RET* activating mutation was clinically thought to be MTC.

183 patients harbored variants of uncertain significance or negative results, according to genetic tests. 20 (10.93%) out of the 183 underwent surgery, and 12 were diagnosed as malignant thyroid tumors, including 11 PTC and 1 MTC.

## 4 Discussion

Presently, the application of NGS-based multi-gene testing and molecular markers in conjunction with cytologic diagnosis has shown promising results in improving the preoperative diagnosis of indeterminate thyroid nodules, consequently decreasing the number of needless surgeries. Multi-gene assays with favorable Sen, Spe, PPV, and NPV are becoming more and more common. In an Indian study, NGS-based panel testing including six genes was performed on FNA biopsy samples from 69 thyroid nodules patients, with a sensitivity of 81.5% (11). Additionally, a study conducted in China included 73 FNA biopsy samples, with a sensitivity of 85.5% by NGS-based panel testing, which included sixteen gene detections (12). While the diagnostic sensitivity of NGS combined with cytology in this study is 89.8% (95% CI: 0.8223-0.9437).

We also found that a significant portion of previous studies carried out up to this point have concentrated on thyroid nodules with cytological indeterminacy, possibly omitting portions of nodules that have been cytopathologically classified as Bethesda I and II. Although these nodules are classified as non-diagnostic or benign on cytopathology, there is a possibility that they may be malignant. In the present research, two patients were cytopathologically classified as Bethesda I; however, our NGS-based multi-gene test of the FNA biopsy samples revealed a *BRAF* ^V600E^ mutation. In addition, the resected nodule samples were eventually surgically pathologically classified as PTC. It was known that NGS-based multi-gene panel testing needed fewer samples than that of cytopathology, validating that the NGS-based multi-gene panel would correctly identify some of the samples that cytopathology was unable to detect.

Additionally, all patients with *BRAF* ^V600E^ who underwent surgery were identified as PTC by surgical pathology, which indicates a sensitivity of 1 (95% CI: 0.9531-1). These findings align with previous research; certain mutations, such as *BRAF* ^V600E^ and *TERT*, exhibit a nearly 100% risk of PTC and are very specific (10, 13). Regardless of the cytopathological Bethesda classification, patients with thyroid nodules who have previously had an FNA biopsy sample tested using an NGS-based multi-gene panel should be alerted that the nodule is PTC if the result is a *BRAF* ^V600E^ mutation.

Single *TERT* promoter variants, *BRAF* ^V600E^ or *RAS* mutations and *TERT* promoter mutations or *TP53* inactivating mutations co-mutations will reduce progression-free survival and are highly associated with aggressive tumor cells and a high rate of recurrence (6, 10, 14–16). The following variants were linked to a poor prognosis in our study: 1 *TERT* promoter, 1 *BRAF* ^V600E^ +*TP53*, 1 *KRAS*+*TP53*, 1 *HRAS*+*TERT*, 1 *BRAF* ^V600E^ +*TERT*. By surgical pathology, 4 of these patients were diagnosed with malignant thyroid nodules, including 2 PTC, 1 FTC, and 1 DHGTC. The surgical pathological diagnosis results confirmed that the aforementioned mutations found by NGS were, in fact, closely associated with malignant thyroid nodules. Patients should be reminded to schedule surgical resection as soon as possible and to think about broadening the scope of surgery when any of the aforementioned high-risk mutations are detected.

RAS is a family of GTP-binding proteins. It is believed that activating mutations in *RAS* play a crucial role in the initiation of follicular thyroid carcinoma (FTC) (17). *RAS* mutations have been reported to occur in 28-68% of FTCs and up to 43% of follicular variant PTCs (18), as well as in 20-25% of follicular adenomas (19). The PPV of *RAS* mutations is approximately 30%, according to numerous studies conducted to date (20, 21). According to the NGS-based multi-gene panel testing, the PPV of *RAS* mutations was 0.3 (95% CI: 0.1078-0.6032) in our cohort of surgical patients, while the PPV of FNA cytopathology was 0.2 (95% CI: 0.0567-0.5098). Of the patients with *RAS* mutations in the cohort of non-surgical patients, 82.35% were classified as Bethesda I/II. This highlights the limitations of predicting the prognosis of TC solely based on *RAS* mutations. *RAS* mutations are therefore regarded as intermediate-risk variants. Both benign and malignant thyroid diseases can have *RAS* mutations. The doctors should carefully evaluate whether surgery or additional follow-up is necessary for the patients with *RAS* mutations according to their other clinical symptoms.

Additionally, *RET* activating mutations, *RET* fusions, and *TP53* inactivating mutations were found by our NGS-based multi-gene panel testing. However, since the patients did not undergo surgery, the benign and malignant nodules could not be identified. According to the aggressiveness of MTCs carrying *RET* mutations research has been conducted to divide *RET* mutations into three risk groups: highest-, high-, and moderate-risk mutations (22, 23). In the aforementioned studies, *RET* ^C634R^, which was detected in our statistical sample, was categorized as high-risk. And *RET* fusions are substantially more common than *NTRK* fusion, *BRAF* ^V600E^ and *RAS* mutations in aggressive tumor behavior, which includes high rates of lymph node and distant metastases (24). Inactivating mutations in *TP53* are the genetic hallmarks of ATC. Recent research discovered that 86% of resected nodules with isolated *TP53* mutations were benign, while 82% of nodules carrying *TP53* mutations along with other gene alterations were cancerous; although the majority of thyroid nodules containing a single *TP53* genetic variant have been shown to be benign adenocarcinomas, there has been speculation that *TP53*-mutant adenocarcinomas could be precursors to malignancies that have not yet differentiated (25). Thus, we thought thyroid nodules containing *TP53* inactivating mutations required special attention, while *RET* activating mutations and RET fusions are closely linked to malignant thyroid nodules.

Among the 183 patients with negative genetic test results or variants of uncertain significance, 20 patients underwent surgery, and 12 patients were diagnosed with malignant thyroid tumors. Of the 12 malignant tumors, 10 presented negative results from genetic testing. It is reasonable to anticipate that an NGS-based multi-gene panel utilizing FNA biopsy samples will yield a negative result or a variant of uncertain significance. Thus, the potential for deterioration needs to be emphasized even in thyroid nodules identified by NGS-based multi-gene probes as carrying non-pathogenic variants. Furthermore, the DNA from HE-stained sections appears to be a useful source of DNA for routine DNA testing (26). In clinical practice, FNA biopsy samples can be obtained for HE staining and cytopathologic analysis. After that, the HE-stained slides are sent for NGS testing, thereby reducing the results of negative or variants of uncertain significance. Concurrently, through the observation of additional clinical indicators, clinicians can ascertain whether patients with negative or variants of uncertain significance require surgical intervention or regular clinical evaluation. The most prevalent clinical indicators at present are the ultrasound-described nodule size, the nodule boundary’s clarity, the nodule’s calcification content, and the type of blood supply of nodules.

**Figure 2:**
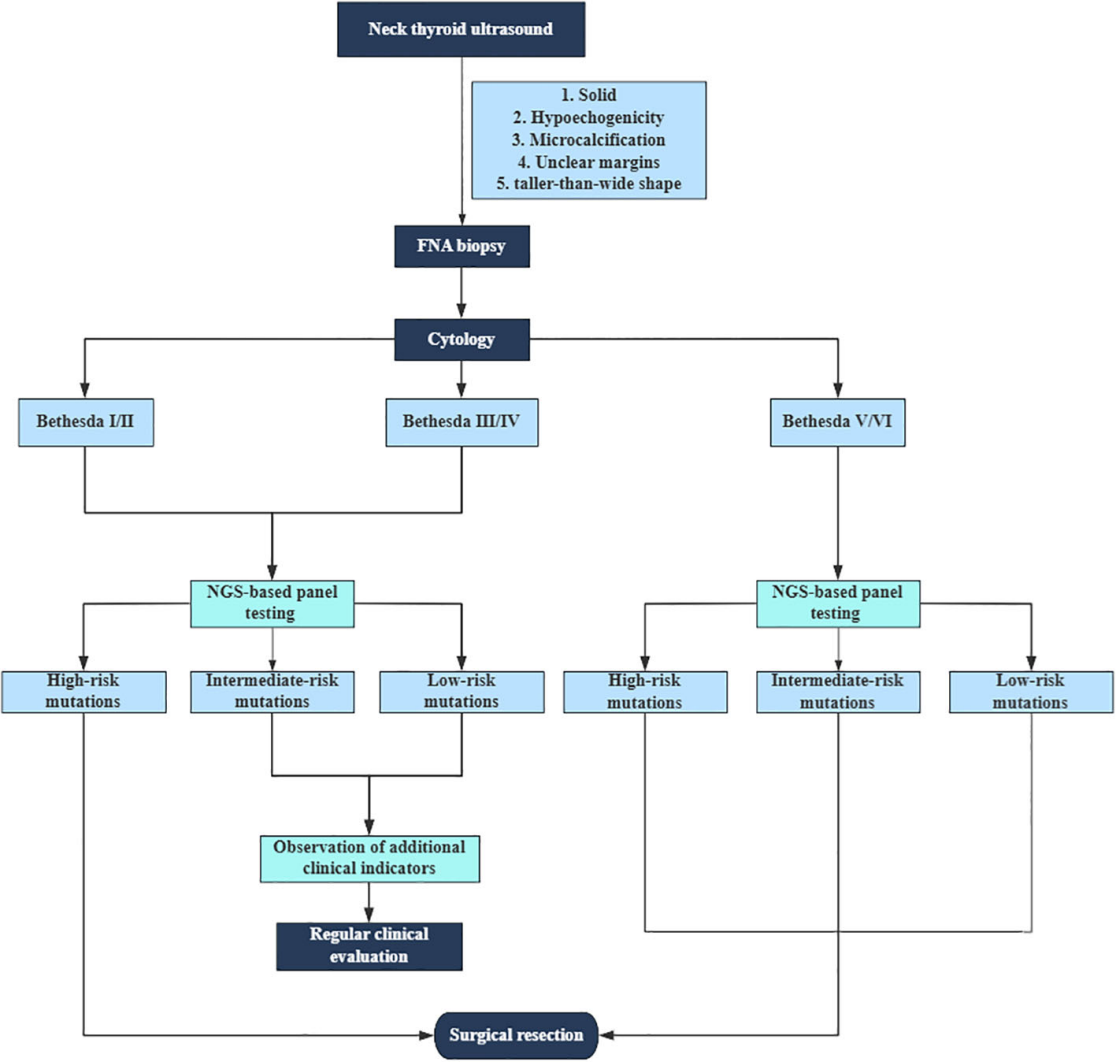
Flowchart of the clinical workflow for the management of thyroid nodules.

There are a few limitations pertinent to this study, with a limited number of genes included in the panel due to cost. The current research about the incidence, metastasis, and prognosis of thyroid cancer indicated that *PIK3CA*, *PTEN*, *CDKN1B*, *TSHR*, and *EIF1AX* were also involved, which should be considered in the multi-gene panel (12, 27). It is important to recognize the limitations of FNA biopsy samples for NGS-based panel testing. The following aspects require particular consideration: tumor heterogeneity, insufficient sampling, false negative risk, sample contamination, and puncture technical issues. In addition, with longer follow-up, more patients’ surgical pathology results may be available (but there was still a reasonable number of cases), making the results more convincing. This study is a single-center clinical trial and showed slightly larger intervention effects than did multi-center trials. However, we were successfully able to identify that the thyroid nodules with *BRAF* ^V600E^, *TERT* promoter mutations, *BRAF*/*RAS* co-mutation with other genes (e.g., *KRAS*+*TP53*, *BRAF* ^V600E^+*TP53*, *HRAS*+*TERT*, *BRAF* ^V600E^+*TERT*), *RET* activating mutations, and *RET* fusions belonged to the high-risk group. The intermediate-risk group includes the *RAS* mutations and *TP53* inactivating mutations, while the non-pathogenic (negative and variants of uncertain significance) mutations identified were classified as low-risk. Our study stratifies and assesses the risk of thyroid nodules based on specific gene mutations, which can offer patients more individualized diagnostic reference recommendations. However, in the traditional genetic test category, the results are expressed as pathogenic or negative. A flow chart was constructed, and a summary of the clinical workflow for the management of thyroid nodules based on cytopathology and NGS-based panel test results was produced (**Figure 2**).

When it comes to risk stratification and the ability to distinguish benign from malignant thyroid nodules, NGS-based multi-gene panel testing has demonstrated diagnostic performance that exceeds expectations (28–30). The current goals of the present research on the molecular characteristics of thyroid nodules are to improve the ability to distinguish between benign and malignant thyroid nodules and to forecast their clinical course. A further step in patient risk stratification is extending the follow-up period for patients with thyroid nodules. Given that the genetic variant map of TC is gradually being shown in greater detail, the diagnostic performance and application of NGS-based multi-gene panel testing are also anticipated.

## Data Availability

The original contributions presented in the study are included in the article/supplementary material. Further inquiries can be directed to the corresponding authors.
